# Use of Outpatient-Derived COVID-19 Convalescent Plasma in COVID-19 Patients Before Seroconversion

**DOI:** 10.3389/fimmu.2021.739037

**Published:** 2021-09-14

**Authors:** Oliver F. Wirz, Katharina Röltgen, Bryan A. Stevens, Suchitra Pandey, Malaya K. Sahoo, Lorna Tolentino, Michelle Verghese, Khoa Nguyen, Molly Hunter, Theo Thomas Snow, Abhay Raj Singh, Catherine A. Blish, Jennifer R. Cochran, James L. Zehnder, Kari C. Nadeau, Benjamin A. Pinsky, Tho D. Pham, Scott D. Boyd

**Affiliations:** ^1^Department of Pathology, Stanford University School of Medicine, Stanford, CA, United States; ^2^Stanford Blood Center, Palo Alto, CA, United States; ^3^ATUM, Newark, CA, United States; ^4^Sean N. Parker Center for Allergy and Asthma Research, Stanford, CA, United States; ^5^Department of Medicine, Division of Infectious Diseases and Geographic Medicine, Stanford University, Stanford, CA, United States; ^6^Chan Zuckerberg Biohub, San Francisco, CA, United States; ^7^Department of Bioengineering, Stanford University, Stanford, CA, United States; ^8^Department of Medicine, Division of Pulmonary, Allergy and Critical Care Medicine, Stanford University, Stanford, CA, United States

**Keywords:** SARS-CoV-2, COVID-19, convalescent plasma for COVID-19 therapy, humoral immune response, antiviral antibodies

## Abstract

**Background:**

Transfusion of COVID-19 convalescent plasma (CCP) containing high titers of anti-SARS-CoV-2 antibodies serves as therapy for COVID-19 patients. Transfusions early during disease course was found to be beneficial. Lessons from the SARS-CoV-2 pandemic could inform early responses to future pandemics and may continue to be relevant in lower resource settings. We sought to identify factors correlating to high antibody titers in convalescent plasma donors and understand the magnitude and pharmacokinetic time course of both transfused antibody titers and the endogenous antibody titers in transfused recipients.

**Methods:**

Plasma samples were collected up to 174 days after convalescence from 93 CCP donors with mild disease, and from 16 COVID-19 patients before and after transfusion. Using ELISA, anti-SARS-CoV-2 Spike RBD, S1, and N-protein antibodies, as well as capacity of antibodies to block ACE2 from binding to RBD was measured in an *in vitro* assay. As an estimate for viral load, viral RNA and N-protein plasma levels were assessed in COVID-19 patients.

**Results:**

Anti-SARS-CoV-2 antibody levels and RBD-ACE2 blocking capacity were highest within the first 60 days after symptom resolution and markedly decreased after 120 days. Highest antibody titers were found in CCP donors that experienced fever. Effect of transfused CCP was detectable in COVID-19 patients who received high-titer CCP and had not seroconverted at the time of transfusion. Decrease in viral RNA was seen in two of these patients.

**Conclusion:**

Our results suggest that high titer CCP should be collected within 60 days after recovery from donors with past fever. The much lower titers conferred by transfused antibodies compared to endogenous production in the patient underscore the importance of providing CCP prior to endogenous seroconversion.

## Highlights

High-titer convalescent plasma can be collected from low-severity outpatients with history of fever and typically within 60 days after symptom cessation. High-titer convalescent plasma should be adminstered to COVID-19 patients before endogenous seroconversion occurs.

## Introduction

The COVID-19 pandemic is exacting a terrible toll on societies and health systems worldwide. Transfusion of COVID-19 convalescent plasma (CCP) containing anti-SARS-CoV-2 antibodies may have therapeutic benefit for COVID-19 patients until more efficacious therapeutics are widely available. CCP is also used as a source for purifying SARS-CoV-2-specific immunoglobulins for more standardized antibody treatment regimens (e.g. anti-coronavirus hyperimmune intravenous immunoglobulin). In the United States, vaccines and therapeutic monoclonal antibodies have been given emergency use authorization by the Food and Drug Administration (FDA), but logistical and financial limitations may limit the use of these interventions, especially in low- and middle-income countries, favoring the continued use of patient-derived antibody-based therapies such as CCP. It is therefore crucial to assess the magnitude and stability of serological responses in CCP donors and define an ideal timeframe for CCP donation. While some studies show SARS-CoV-2-specific B cells and detectable levels of SARS-CoV-2-specific antibodies for several months after infection (1–3), others have shown that antibody levels begin to decrease as early as one month after symptom onset, especially in less severely ill outpatients (4, 5). Although CCP efficacy in all COVID-19 patients is equivocal (6–8), recent studies suggest that high-titer CCP administered to patients early in disease course may be protective (9–12), a practice also recently recommended by the FDA (13). Since the majority of SARS-CoV-2-infected individuals, and hence also potential CCP donors, are mildly ill outpatients, we have sought to determine the patient characteristics associated with higher antibody titers in these individuals. Prior studies have lacked detailed time course data for analyzing the kinetics of antibodies derived from CCP in recipients, and for comparing the therapeutic antibody quantities to those derived from the patient’s own humoral immune response early during the disease course, to better understand the potential benefits of early transfusion.

## Methods

### Clinical Specimens

Venipuncture blood samples from 93 COVID-19 convalescent plasma (CCP) donors from the San Francisco Bay Area in California who donated CCP at Stanford Blood Center from 4/14/2020 to 8/25/2020, as well as from 16 COVID-19 patients admitted to Stanford Hospital were collected in sodium heparin- or K_2_EDTA-coated vacutainers and plasma was used for serology testing, N-antigenemia testing, and rRT-PCR detection of RNAemia. Plasma samples were stored at 4°C (short-term) or -80°C (long-term). For three transfused COVID-19 patients, the sampling timepoints were not ideal to assess whether the transfused CCP influenced the recipient’s plasma antibody levels (shown in **Supplementary Figure 1**). Retrospective chart review was performed on all COVID-19 patients admitted to Stanford Hospital. This study was approved by the Stanford University Institutional Review Board (Protocols IRB-48973, IRB-55689, and IRB-13952). All patients were transfused CCP as a part of the National Convalescent Plasma Expanded Access Protocol sponsored by the Mayo Clinic and approved by the Stanford University Institutional Review Board (Protocol IRB-56100).

### ELISA to Detect Anti-SARS-CoV-2 Antibodies in Plasma Samples

The ELISA protocol used in the present study was described by Röltgen et al. (4). In brief, ELISA plates were coated with SARS-CoV-2 spike RBD, S1, or N protein at a concentration of 0.1 μg per well (0.025 μg per well for the N protein IgG assay). Plasma samples from CCP donors and COVID-19 patients were incubated at a dilution of 1:100 for 1 hour. Anti-SARS-CoV-2 IgA, IgG, and IgM antibodies were detected using HRP (horseradish peroxidase) conjugated goat anti-human IgG (γ-chain specific, catalog no. 62-8420, Thermo Fisher, 1:5,000 dilution), IgM (μ-chain specific, catalog no. A6907, Sigma, 1:5,000 dilution), or IgA (α-chain specific, catalog no. P0216, Agilent, 1:2,000 dilution). Development was done using 3,3′,5,5′-Tetramethylbenzidine (TMB) substrate and optical density (OD) at 450 nm was measured with a microplate reader and blank values were subtracted from values obtained for plasma samples. Seroconversion for each isotype/protein assay was defined as values above mean ELISA ODs of 94 negative control samples from healthy blood donors collected before the pandemic plus three times their standard deviation (mean + 3 SD). All samples were tested twice in independent experiments.

### Competition ELISA to Detect Antibodies That Block Binding of ACE2 to RBD

The protocol for the competition ELISA procedure used here was recently described by Röltgen et al. (4) In brief, plates were coated with SARS-CoV-2 spike RBD protein and then incubated with plasma samples at a dilution of 1:10 for 1 hour at room temperature. Then, recombinant ACE2 joined to a mouse IgG2a Fc (ACE2-mFc) at 0.5 µg/mL was added to the plasma sample for another 45 minutes. After washing, RBD-ACE2-mFc was detected using horseradish peroxidase conjugated anti-mouse IgG. ELISA plates were developed and measured as described above. A positive and a negative quality control (Access SARS-CoV-2 IgG QC, QC1-QC2, catalog no. C58964, Beckman Coulter) was included on each plate. OD values were converted to ‘*% ACE2 blocking*’ using the following formula: *% ACE2 blocking* = 100*(1-(sample OD - 0.2)/(QC1 OD – 0.2)), taking into account the background noise of the assay of 0.2 which was determined by testing negative control plasma samples that were collected before the pandemic. All samples were tested two times in independent experiments.

### Real-Time PCR for Detection of SARS-CoV-2 RNA in Plasma Samples

The protocol for detection of SARS-CoV-2 RNA in plasma was performed based on a published rRT-PCR assay targeting the envelope (*E*) gene (14, 15). RNA was isolated from 400 μL of EDTA-anticoagulated plasma using Qiagen EZ1 Virus Mini Kit v2.0 (Qiagen German-town, MD). Ct values of positive tests with this assay normally range from Ct <20 to 45 cycles. Testing of plasma samples with a Ct value of 40 or higher were tested again to ensure reproducibility of the positive result. No viral culture was performed as part of this study, therefore, presence of SARS-CoV-2 in tested plasma was defined as RNAemia.

### Antigen Detection

SARS-CoV-2 nucleocapsid antigen was quantified using S-PLEX Direct Detection Assay, S-PLEX SARS-CoV-2 N Kit (Catalog #K150ADHS, Meso Scale Discovery [MSD], Rockville, MD), according to manufacturer’s protocol. Raw signal was converted to a concentration based on linear regression to the 7-point calibration curve. Cut off for positivity was calculated as the mean value of 40 pre-pandemic plasma samples plus three times the standard deviation.

### Statistics

GraphPad Prism version 8.4.1 software (GraphPad Software, San Diego, California, USA) was used to visualize data, analyze for differences in antibody responses and N-antigenemia levels between different timepoints to carry out linear regression of % RBD-ACE2 blocking and antibody titers. Ordinary one-way ANOVA test and Kruskal-Wallis test with Dunn’s multiple comparison test was used to compare more than two groups when samples either followed, or did not follow Gaussian distribution, respectively. Unpaired t-test was used to compare IgG levels and % RBD-ACE2 blocking in samples from symptom positive *versus* negative patients, while no correction for multiple comparison was performed. Goodness of fit for linear regression analyses was reported as the coefficient of determination (R2). Correlation between antibody OD450 values, RNAemia, and RBD-ACE2 blocking assay OD450 values were calculated as Spearman correlations with the R cor function. Two-sided tests with p<0.05 were considered as statistically significant.

## Results

### Time After Recovery and Symptoms Correlate to Humoral Immune Response in Mildly Ill COVID-19 Convalescent Plasma Donors

**Figure 1:**
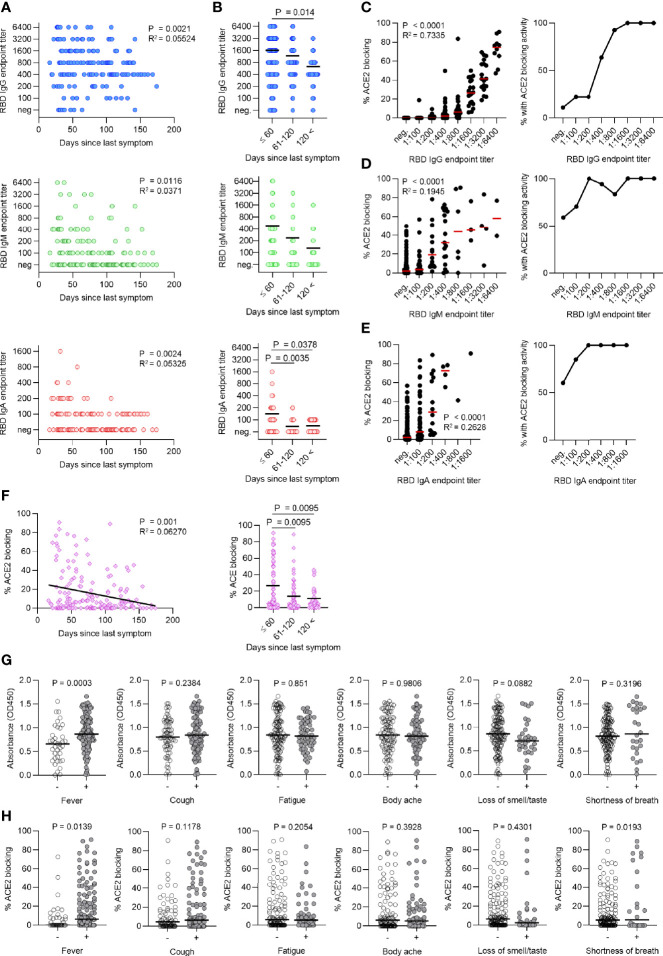
Time after recovery and symptoms correlate to humoral immune response in mildly ill COVID-19 convalescent plasma donors. **(A)** Titers of SARS-CoV-2 RBD-specific IgG, IgM, and IgA in the plasma of 93 COVID-convalescent plasma (CCP) donors (172 individual samples) decrease over time after symptom cessation. **(B)** Antibody titers begin to decrease after 120 days. **(C–E)** Titers of IgG **(C)**, IgM **(D)**, and IgA **(E)** were correlated to RBD-ACE2 blocking activity (in %, left panel). The percentage of cases with any detectable RBD-ACE2 blocking activity is shown for each titer (right panel). **(F)** RBD-ACE2 blocking capacity (in %) over time shown for all cases (left panel) and separated in bins of 60 days (right panel). **(G, H)** Comparison of RBD-specific IgG titers **(G)** (Absorbance, OD450) and RBD-ACE2 blocking activity **(H)** in symptom positive *versus* negative cases for most common reported symptoms.

We studied the SARS-CoV-2-specific humoral immune response in 172 CCP samples collected from 93 non-hospitalized outpatients (**Table 1**). In contrast to earlier studies (4, 5), samples were collected up to 174 days after convalescence. We measured IgM, IgA, and IgG levels in the plasma of these donors against the SARS-CoV-2 Spike S1 region, receptor binding domain (RBD) and nucleocapsid antigen (N) using laboratory-developed ELISAs. Anti-RBD titers decreased with time after symptom cessation (**Figure 1A**). Antibody levels were highest in CCP donations collected within two months after symptom resolution and were markedly decreased after 120 days (**Figure 1B**). Similarly, antibody titers waned with time for anti-S1 (**Supplementary Figure 2**). Analysis of individual donors with four or more donation timepoints clearly revealed that antibody signals consistently decreased over time (**Supplementary Figure 3**).

**Table 1:** Non-hospitalized CCP donor demographics and clinical characteristics.

Characteristics		n = 93
Age, median (IQR)		48 (35-56)
Sex	Female	28 (30.1%)
	Male	65 (69.9%)
Symptom, N of individuals (% present)	Fever	70 (75.3 %)
	Cough	55 (59.1 %)
	Body ache	36 (38.7 %)
	Lethargy/Tiredness/Fatigue	29 (31.2 %)
	Loss of smell/taste	20 (21.5 %)
	Headache	19 (20.4 %)
	Dyspnea	18 (19.4 %)
Number of timepoints, N of individuals (% present)	1 timepoint	55 (59.1%)
	2 timepoints	18 (19.4%)
	3 timepoints	8 (8.6%)
	4 timepoints	8 (8.6%)
	>4 timepoints	4 (4.3%)

CCP, COVID-19 convalescent plasma; IQR, interquartile range. The 12 samples from six hospitalized CCP donors, as well as three samples from donors with no symptom description, were excluded.

Viral spike RBD interaction with human angiotensin-converting enzyme 2 (ACE2) initiates SARS-CoV-2 entry into host cells. We performed an RBD-ACE2 blocking ELISA to measure the functional activity of plasma antibodies to block RBD-ACE2 interaction. CCP donor anti-RBD IgG levels were positively correlated to RBD-ACE2 blocking capacity and all samples with titers of at least 1:1600 exhibited RBD-ACE2 blocking activity, while only a subset of samples with lower titers showed RBD-ACE2 blocking activity (**Figure 1C**). We also measured anti-RBD IgM and IgA titers, which showed weaker correlations to RBD-ACE2 blocking (**Figures 1D, E**). Similarly, RBD-ACE2 blocking capacity was significantly higher in CCP samples collected within 60 days post symptom (**Figure 1F**). Together, this further emphasized the importance of CCP donations early after recovery.

Identifying CCP donor factors associated with high antibody titers would contribute to more efficient donor recruitment strategies. We therefore explored whether certain symptoms reported in our cohort of mildly ill outpatients (**Table 1**) correlated with anti-RBD IgG levels (**Figure 1G**) and RBD-ACE2 blocking capacity (**Figure 1H**). Interestingly, fever was the only symptom that distinguished CCP donors with higher levels of anti-RBD IgG and RBD-ACE2 blocking activity (**Figures 1G, H**). Similarly, increased anti-S1 and anti-N IgG antibodies were found in patients with fever (data not shown).

### SARS-CoV-2-Specific Antibody Levels, Viral N-Antigenemia and RNAemia in COVID-19 Patients Before and After Convalescent Plasma Therapy

**Figure 2:**
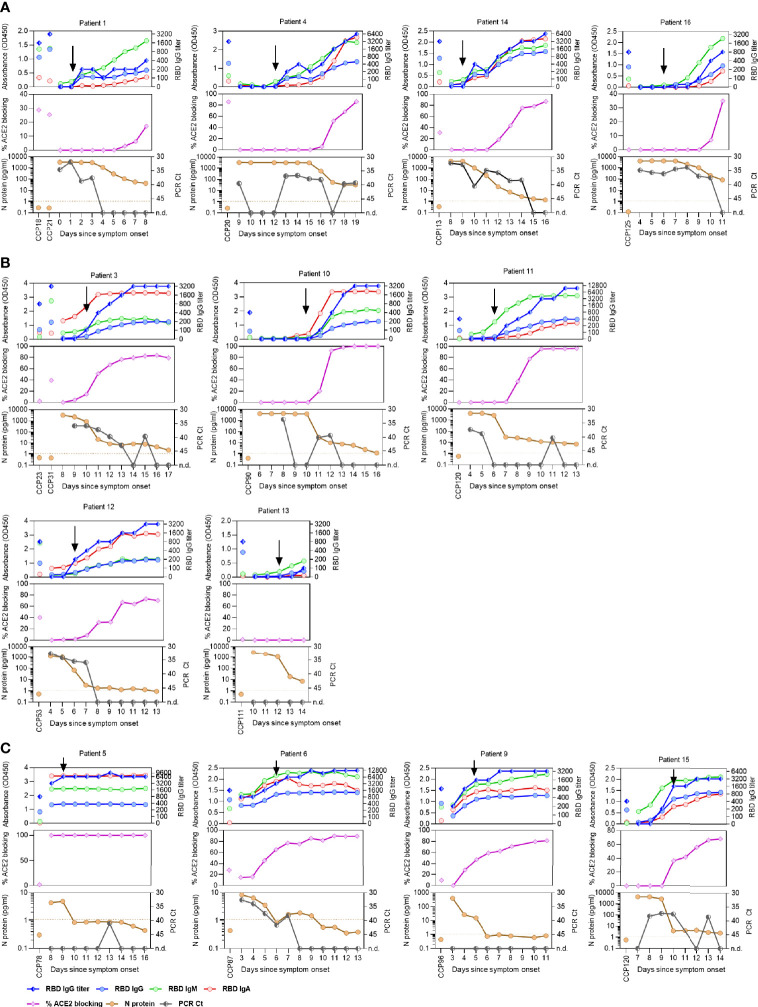
SARS-CoV-2-specific antibody titers, viral N-antigenemia and RNAemia in COVID-19 patients before and after convalescent plasma therapy. Absorbance level of SARS-CoV-2 RBD-specific IgG, IgM, and IgA (1:100 diluted plasma samples), titer of RBD-specific IgG, ACE2 blocking activity (in %), as well as levels of N-antigenemia, and RNAemia are shown for patients that received COVID-19 convalescent plasma (CCP) before **(A)**, during **(B)**, or after seroconversion **(C)**. Timepoints of CCP transfusion are indicated by black arrows.

Several reports showed the benefits of CCP transfusions at early times during the disease course for patients infected with SARS-CoV-2 (9–12, 16), likely because CCP was transfused before these patients seroconverted. We therefore aimed to understand the patients’ immune response at the time of transfusion, analyze potential immediate biological effect of CCP transfusions, and compare these to the endogenous response. To address this, we measured anti-SARS-CoV-2 antibodies, RBD-ACE2 blocking functional antibody levels, viral RNAemia and N-antigenemia in a group of 16 COVID-19 inpatients prior to CCP transfusion and daily for up to one week thereafter (Patient information, **Table 2**). Increases in antibody levels one day after CCP transfusion were observed in four COVID-19 patients who had not yet seroconverted and who received CCP units with high levels of specific IgG antibodies (**Figure 2A**). Anti-RBD IgG antibody titer increased immediately after the transfusion, followed by a plateau or slight decrease; we attribute this serological response to the CCP transfusion.

**Table 2:** Demographics and clinical characteristics of 16 CCP-treated COVID-19 patients.

Characteristics		Admitted, non-ICU (n = 4)	Admitted, ICU (n = 8)	Admitted, ICU, Deceased (n = 4)
Patient number		1, 13, 15, 16	4, 7, 8, 9, 10, 11, 12, 14	2, 3, 5, 6
Age, mean		49	51	61
Sex (%)	Female	3 (75)	2 (25)	1 (25)
	Male	1 (25)	6 (75)	3 (75)
Symptom, N of individuals (% present)	Dyspnea	3 (75)	6 (75)	2 (50)
	Fever	2 (50)	5 (62.5)	2 (50)
	Cough	3 (75)	2 (25)	3 (75)
	GI	3 (75)	3 (37.5)	1 (25)
	Myalgia	2 (50)	2 (25)	1 (25)
	Chills	2 (50)	1 (12.5)	1 (25)
	Fatigue	0 (0)	1 (12.5)	1 (25)
	Confusion	0 (0)	2 (25)	0 (0)
	Headache	1 (25)	0 (0)	0 (0)
Comorbidities, N of individuals (% present)	Obesity	2 (50)	4 (50)	1 (25)
	Diabetes mellitus	2 (50)	4 (50)	3 (75)
	Hypertension	1 (25)	5 (62.5)	4 (100)
	Asthma	1 (25)	2 (25)	0 (0)
Mechanical ventilation (%)		0 (0)	4 (50)	4 (100)
Admission, days post symptoms, mean		5	4	7
Length of hospital stay, mean		13	29	38
CCP therapy, days post symptoms, mean		7	8	8
Seroconverted before CCP therapy, N of individuals (%)		2 (50)	6 (75)	4 (100)

CCP, COVID-19 convalescent plasma; ICU, intensive care unit.

In contrast, CCP was transfused in nine patients either near the timepoint of anti-RBD IgG seroconversion (**Figure 2B**), or who already developed high antibody titers and RBD-ACE2 blocking activity (**Figure 2C**). Here, it is difficult to separate the serological effect of the transfused CCP from the patients’ own response. For three patients, the sampling timepoints were not suitable to assess whether the transfused CCP influenced the recipient’s plasma antibody levels (**Supplementary Figure 1**). Similar results were found when we measured titers of antibodies specific for Spike S1 region and N-antigen in these patients (**Supplementary Figure 4**). With the plasma dilution used in our experiment, two patients reached maximal RBD-ACE2 blocking activity between one and two weeks after symptom onset, as a result of their own serological response.

With the aim to assess an effect of the transfused plasma, N-antigen and viral RNA levels in the blood were measured to estimate viral load. N-antigenemia was found in 93.75% (15/16) and RNAemia in 75% (12/16) of patients (**Figure 2**, **Supplementary Figure 1**). Inversely correlating with their serological responses, N-antigenemia and RNAemia were reduced in all patients over the course of their illness (**Figure 2**, **Supplementary Figure 1**). Interestingly, two patients (1 and 14) who received CCP before seroconversion showed reduced RNAemia immediately following CCP transfusion (**Figure 2A**), potentially supporting efficacy of early CCP administration. Over the course of the study period, N-antigenemia was becoming undetectable for 40% (6/15) of the patients while RNAemia resolved in 75% (9/12) of previously positive patients. The persistence of low levels of declining viral RNA and protein in the blood of seroconverted patients could be due to the antibodies not yet having achieved the concentrations needed to fully bind and opsonize the remaining viral proteins in the body. Antibody titers negatively correlated to N-antigen levels in these patients (**Supplementary Figure 5A**) and level of N-antigen at timepoint of transfusion distinguished hospitalized patients where CCP was given before seroconversion (**Supplementary Figure 5B**).

## Discussion

Here, we studied the immune response to SARS-CoV-2 infection in mildly ill outpatients that donated COVID-19 convalescent plasma (CCP). High titer plasma important for transfusion was mainly found within 60 days after symptom cessation and in patients that had fever. Furthermore, we analyzed whether transfused CCP can be detected and has a direct effect on viral antigens and viral RNA levels in 16 hospitalized COVID-19 patients. An effect was found only in those individuals that did not seroconvert yet.

Logistical and financial limitations may still limit the use of vaccines and therapeutic monoclonal antibodies, especially in low- and middle-income countries, favoring the continued use of patient-derived antibody-based therapies such as CCP. Here, we aimed to identify donor factors associated with high antibody titers to improve CCP donor recruitment strategies. For this, we first assessed titers of SARS-CoV-2 Spike-RBD, -S1, and N-specific antibodies in relation to symptom cessation.

Antibody titers continuously waned after symptoms ended with most marked decrease after 120 days. Our data indicated that CCP collected within 60 days after symptom resolution is most likely to maximize antibody levels for transfusion. While we studied mildly ill outpatient which make up the majority of SARS-CoV-2 infected individuals and hence potential blood donors, a similar timeframe for collecting high titer plasma was also suggested for more severely ill patients (17). In addition, we also measured the functional activity of antibodies to block RBD-ACE2 interaction. Results from the here used RBD-ACE2 blocking assay closely correlate with a SARS-CoV-2 pseudotyped virus neutralization assay (4). RBD-ACE2 blocking activity was found in all plasma units with an anti-RBD IgG titer of at least 1:1600. This is concordant with a recent study where similar IgG titers were associated with efficient virus neutralization (5). Since anti-RBD antibodies of IgM and IgA isotypes showed weaker correlation to RBD-ACE2 blocking activity, we concluded that anti-RBD IgG titers were the best correlate for virus-neutralizing activity.

In our donor cohort consisting of mildly ill outpatients, we found that fever was the only symptom correlating to higher antibody levels. While dyspnea was relatively rare among our studied cohort (19.4%), a recent study including CCP donors that were more severely ill found increased antibody levels among patients with dyspnea (5).

Several trials have studied the benefit of high titer CCP transfusions on COVID-19 outcome with median duration of symptoms at day of transfusion ranging from 3 to 30 days (10, 18–20). The importance of transfusions at early times during the disease course has been noted for patients infected with SARS-CoV-2 (9–12, 16), and also for SARS-CoV (21). The benefits from CCP transfusion are likely to be greatest for patients who have not yet seroconverted, if the patient’s endogenous neutralizing antibody response is greater in magnitude than the transfused antibody quantity. It is therefore critically important to understand the patients’ immune response at the time of transfusion, analyze the immediate biological effect of CCP transfusions, and compare these to the endogenous response. Sampling COVID-19 patients before and daily up to one week after the CCP transfusion allowed to detect transfused antibodies in four of the studied COVID-19 patients that did not seroconvert yet. At later times afterwards, a rapid increase in antibody levels was seen, very likely reflecting the patients’ own endogenous antibody production and seroconversion. At the same time when we observed the endogenous antibody increase, N-antigenemia and RNAemia resolved in most patients. While the four patients who received CCP before seroconversion recovered from COVID-19, our analysis was not designed to evaluate the efficacy of CCP transfusions. These findings, however, are in line with previous studies showing reduced fatal disease outcomes when CCP was administered early after symptom onset and before seroconversion (10, 11, 16, 21). In contrast, little clinical effect was seen when CCP was transfused more than 14 days after symptom onset (12, 18, 22, 23), likely because at this timepoint the patients already seroconverted with high antibody levels (4). The data are consistent with a role for early CCP administration as a bridging therapy until the patient mounts their own humoral immune response. Standardized serological testing, as opposed to temporal assessment of symptomatology, would be a more mechanistically supported approach to determine patient eligibility for CCP administration.

Limitations of this study include the relatively low number of CCP-treated COVID-19 patients and non-seroconverted patients at time of transfusion. Because most patients seroconvert during infection, the small volume of a unit of CCP (200-300 ml) compared to total plasma volume of patients make it difficult to detect increases in specific antibodies after transfusion in seroconverted patients. We note that we studied outpatient CCP donors, who may have had lower antibody levels compared to inpatients (4).

In this study, we demonstrated that anti-SARS-CoV-2 antibody levels and RBD-ACE2 blocking ability in plasma from outpatient donors were highest within the first two months after symptom resolution, strongly favoring CCP collection early after donor recovery. Donors who had fever during infection had elevated anti-SARS-CoV-2 antibody levels; this criterion may help CCP donor outreach strategies to identify donors with high antibody levels. We showed that increased antibody levels after CCP transfusion were only detected in patients who had not seroconverted at the time of administration, providing a mechanistic basis that could explain why the clinical benefit of CCP therapy appears to be greatest in recipients who are treated soon after symptom onset. In our view, transfusion prior to the patient’s own seroconversion should be considered the relevant clinical goal, informed by rapid serological testing in evaluating the potential benefit of convalescent plasma transfusion in individual patients. This study was performed before the widespread occurrence of viral variants. As new variants continue to emerge, the inter- and intra-strain effectiveness of CCP transfusion should be assessed. Further efforts should be directed at studying the efficacy of CCP administration in COVID-19 patients who have already seroconverted but are still early in the disease course. Use of CCP in immunocompromised patients warrants further study, as this group may stand to benefit the most from the treatment (24, 25).

## Data Availability Statement

The original contributions presented in the study are included in the article/**Supplementary Material**. Further inquiries can be directed to the corresponding authors.

## Ethics Statement

The studies involving human participants were reviewed and approved by Stanford University Institutional Review Board. Written informed consent for participation was not required for this study in accordance with the national legislation and the institutional requirements.

## Author Contributions

OW, TP, and SB conceptualized and designed the study. OW, KR, MS, MV, and KN performed the experiments. OW, KR, BS, SP, MS, LT, MV, KN, TS, and AS collected data and/or contributed samples/reagents or EHR processing methods. CB, JC, JZ, KN, and BP provided intellectual contributions throughout the study. OW and TP performed statistical analyses. OW, TP, and SB analyzed the data. OW, KR, TP, and SB wrote the manuscript. All authors contributed to the article and approved the submitted version.

## Funding

This work was supported by NIH/NIAID R01AI127877 (SB), NIH/NIAID R01AI130398 (SB), NIH 1U54CA260517 (SB), an endowment to S.D.B. from the Crown Family Foundation, an Early Postdoc. Mobility Fellowship Stipend to OW from the Swiss National Science Foundation (SNSF), and a Coulter COVID-19 Rapid Response Award to JC and SB. This work was also supported by MesoScale Diagnostics (MSD), who provided the S-PLEX SARS-CoV-2 N Kits, BioTek 405 Select automated 96-well plate washer, and MESO^®^ SECTOR S 600 Reader used in this study. MSD had no role in study design, data collection and analysis, decision to publish, or preparation of the manuscript.

## Conflict of Interest

SB has consulted for Regeneron, Sanofi, and Novartis on topics unrelated to this study, and owns stock in AbCellera. KN reports grants from National Institute of Allergy and Infectious Diseases (NIAID), Food Allergy Research & Education (FARE), End Allergies Together (EAT); National Heart, Lung, and Blood Institute (NHLBI), and National Institute of Environmental Health Sciences (NIEHS). KN is Director of the FARE and World Allergy Organization (WAO) Center of Excellence at Stanford University; Advisor at Cour Pharmaceuticals; Cofounder of Before Brands, Alladapt, Latitude, and IgGenix; National Scientific Committee member for the Immune Tolerance Network (ITN) of NIAID; recipient of a Research Sponsorship from Nestle; Consultant and Advisory Board Member at Before Brands, Alladapt, IgGenix, NHLBI, and ProBio; and Data and Safety Monitoring Board member at NHLBI. CB is on the board of Catamaran Bio. Author MH was employed by company ATUM.

The remaining authors declare that the research was conducted in the absence of any commercial or financial relationships that could be construed as a potential conflict of interest.

## Publisher’s Note

All claims expressed in this article are solely those of the authors and do not necessarily represent those of their affiliated organizations, or those of the publisher, the editors and the reviewers. Any product that may be evaluated in this article, or claim that may be made by its manufacturer, is not guaranteed or endorsed by the publisher.

## Supplementary Material

Supplementary Figure 1 Measurements in three additional COVID-19 patients. Titers of SARS-CoV-2 RBD-specific IgG, IgM, and IgA, RBD-specific IgG titers, RBD-ACE2 blocking activity (in %), as well as levels of N-antigenemia, and RNAemia are shown for three patients for whom available sample timepoints were not suitable to assess whether the patients had seroconverted before CCP transfusion. Timepoint(s) of CCP transfusion are indicated by black arrows.

Supplementary Figure 2 Titers of SARS-CoV-2 RBD-specific, S1-specific and N-specific IgG, IgM, and IgA antibodies. Titers of SARS-CoV-2 RBD-specific, S1-specific and N-specific IgG, IgM, and IgA antibodies (Absorbance at OD450) plotted *versus* timepoint post symptom cessation.

Supplementary Figure 3 Titers of SARS-CoV-2 RBD-specific, S1-specific and N-specific IgG, IgM, and IgA antibodies for individual donors with multiple timepoints. Titers of SARS-CoV-2 RBD-specific, S1-specific and N-specific IgG, IgM, and IgA antibodies (Absorbance at OD450) plotted versus timepoint post symptom cessation for a selection of donors with > 3 samples.

Supplementary Figure 4 SARS-CoV-2 S1/N-specific antibody titers in COVID-19 patients who received convalescent plasma. Levels of SARS-CoV-2 S1/N-specific IgG, IgM, and IgA antibodies (Absorbance at OD450) are shown for patients who received COVID-convalescent plasma (CCP) before **(A)**, during **(B)**, or after seroconversion **(C)**. **(D)** Shows three patients for whom available sample timepoints were not suitable to assess if patients already seroconverted before CCP transfusion. Timepoint(s) of CCP transfusion indicated by black arrow.

Supplementary Figure 5 N-antigenemia levels negatively correlate with developing immune response. **(A)** Viral N-antigenemia levels for all samples from COVID-19 patients correlated with (from left to right) RBD-specific IgG titers, RBD-specific IgG Absorbance OD450, N-specific IgG Absorbance OD450, and RBD-ACE2 blocking capacity. Reduced N-antigenemia in samples correlated with developing immune response. **(B)** Levels of N-antigenemia in collected plasma from COVID-19 patients distinguished patients that received CCP before, during or after seroconversion.
