# Supplementary figures and images for: Use of Outpatient-Derived COVID-19 Convalescent Plasma in COVID-19 Patients Before Seroconversion
Source: Front Immunol. 2021 Sep 14;12:739037. doi: 10.3389/fimmu.2021.739037 (PMC8477649; doi:10.3389/fimmu.2021.739037)

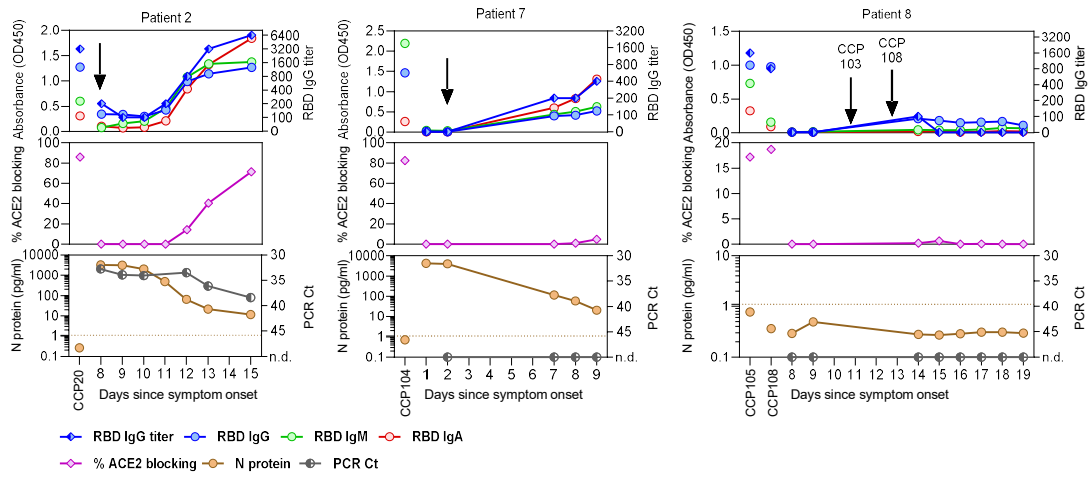

Supplementary Figure 1

Supplement: Supplementary Figure 1 — Measurements in three additional COVID-19 patients. Titers of SARS-CoV-2 RBD-specific IgG, IgM, and IgA, RBD-specific IgG titers, RBD-ACE2 blocking activity (in %), as well as levels of N-antigenemia, and RNAemia are shown for three patients for whom available sample timepoints were not suitable to assess whether the patients had seroconverted before CCP transfusion. Timepoint(s) of CCP transfusion are indicated by black arrows. [file Image_1.pdf]

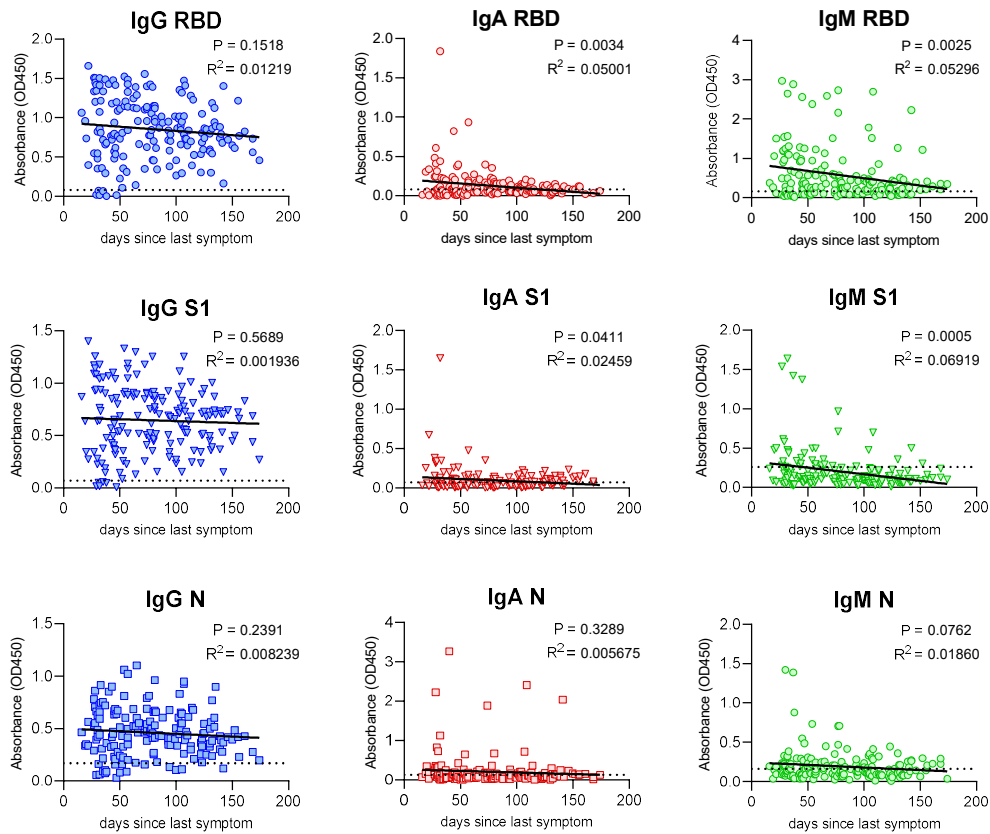

**Supplementary Figure 2**

Supplement: Supplementary Figure 2 — Titers of SARS-CoV-2 RBD-specific, S1-specific and N-specific IgG, IgM, and IgA antibodies. Titers of SARS-CoV-2 RBD-specific, S1-specific and N-specific IgG, IgM, and IgA antibodies (Absorbance at OD450) plotted versus timepoint post symptom cessation. [file Image_2.pdf]

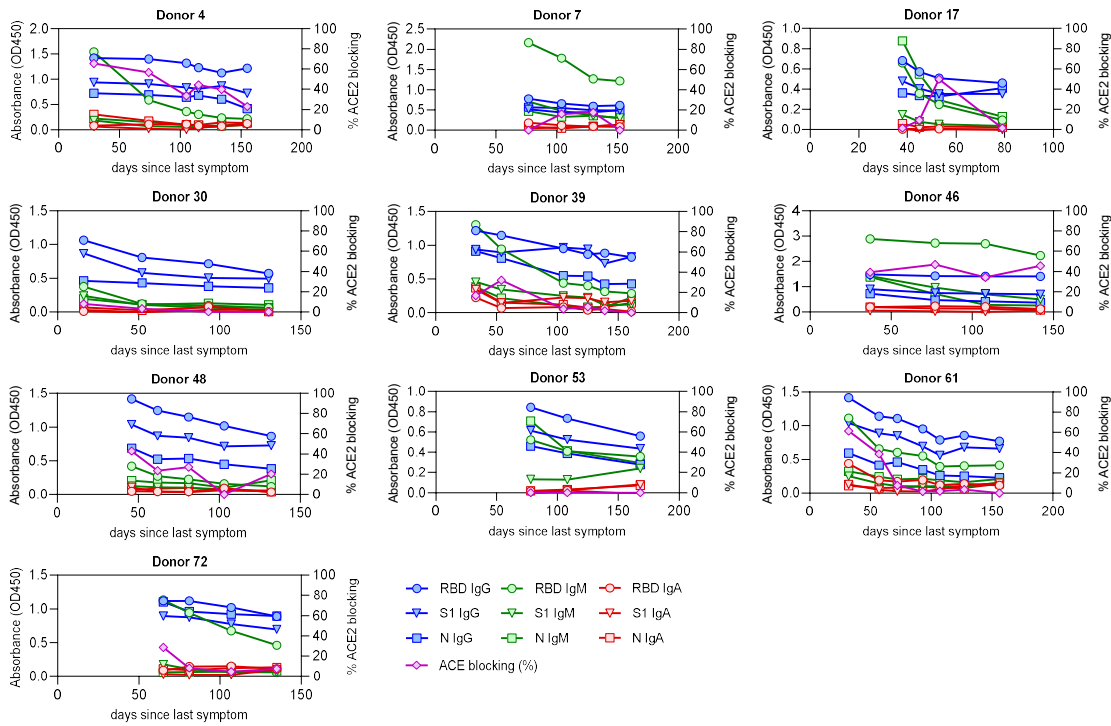

**Supplementary Figure 3**

Supplement: Supplementary Figure 3 — Titers of SARS-CoV-2 RBD-specific, S1-specific and N-specific IgG, IgM, and IgA antibodies for individual donors with multiple timepoints. Titers of SARS-CoV-2 RBD-specific, S1-specific and N-specific IgG, IgM, and IgA antibodies (Absorbance at OD450) plotted versus timepoint post symptom cessation for a selection of donors with > 3 samples. [file Image_3.pdf]

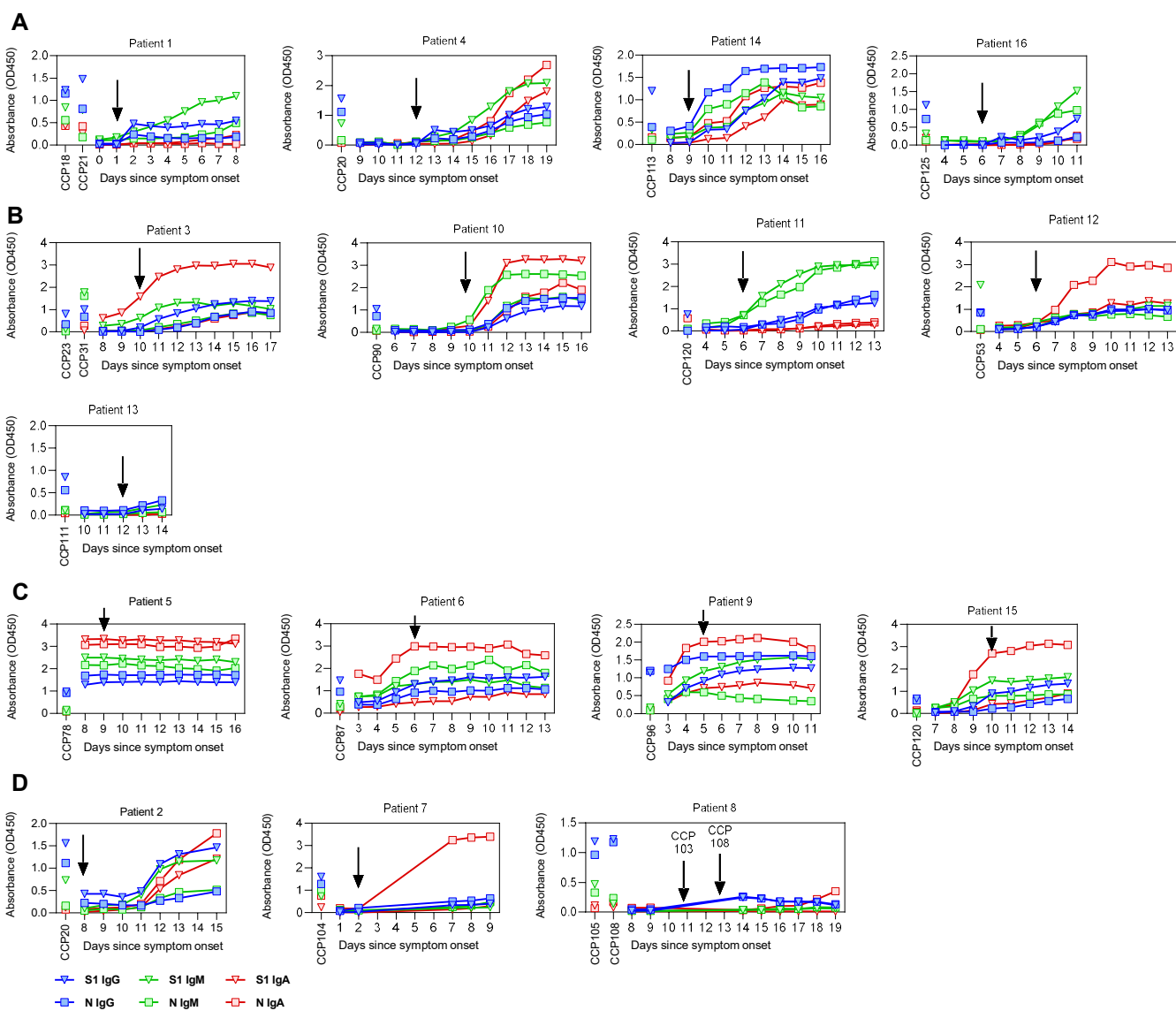

Supplementary Figure 4

Supplement: Supplementary Figure 4 — SARS-CoV-2 S1/N-specific antibody titers in COVID-19 patients who received convalescent plasma. Levels of SARS-CoV-2 S1/N-specific IgG, IgM, and IgA antibodies (Absorbance at OD450) are shown for patients who received COVID-convalescent plasma (CCP) before (A), during (B), or after seroconversion (C). (D) Shows three patients for whom available sample timepoints were not suitable to assess if patients already seroconverted before CCP transfusion. Timepoint(s) of CCP transfusion indicated by black arrow. [file Image_4.pdf]

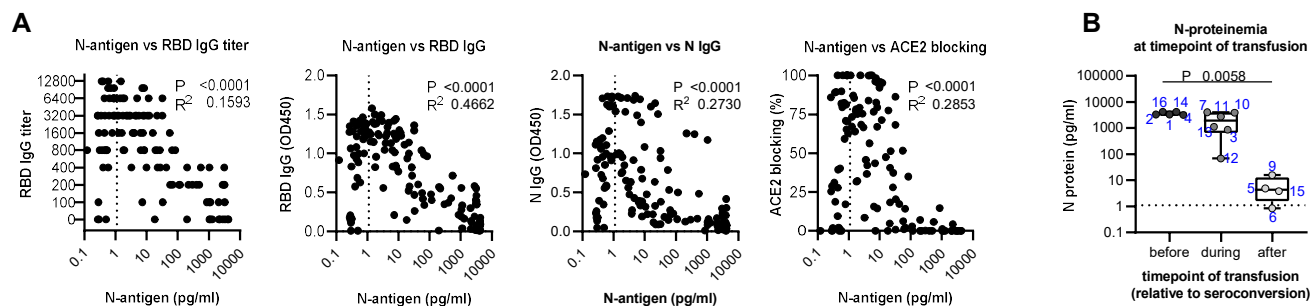

Supplementary Figure 5

Supplement: Supplementary Figure 5 — N-antigenemia levels negatively correlate with developing immune response. (A) Viral N-antigenemia levels for all samples from COVID-19 patients correlated with (from left to right) RBD-specific IgG titers, RBD-specific IgG Absorbance OD450, N-specific IgG Absorbance OD450, and RBD-ACE2 blocking capacity. Reduced N-antigenemia in samples correlated with developing immune response. (B) Levels of N-antigenemia in collected plasma from COVID-19 patients distinguished patients that received CCP before, during or after seroconversion. [file Image_5.pdf]
